# Cotton-like micro- and nanoscale poly(lactic acid) nonwoven fibers fabricated by centrifugal melt-spinning for tissue engineering

**DOI:** 10.1039/c7ra07453k

**Published:** 2018-01-30

**Authors:** Hongli Zhou, Yufeng Tang, Zongliang Wang, Peibiao Zhang, Qingsan Zhu

**Affiliations:** Department of Orthopedics, China-Japan Union Hospital, Jilin University 126 Xiantai Street Changchun 130033 PR China zhuqs@jlu.edu.cn +86 431 85262058 +86 431 85262058; Key Laboratory of Polymer Ecomaterials, Changchun Institute of Applied Chemistry, Chinese Academy of Sciences Changchun 130022 PR China zhangpb@ciac.ac.cn; Department of Traumatology, Qianfoshan Hospital of Shandong Province 250000 PR China

## Abstract

Biodegradable materials in the form of nonwoven fibers have attracted increasing attention for tissue engineering applications because they offer large surface areas and interconnected networks. In this study, cotton-like nonwoven poly(lactic acid) (PLA) fibers were successfully fabricated by centrifugal melt-spinning. The effects of centrifugal speed and secondary melt-spinning processing on the morphology, mechanical properties, and cell compatibility of the fibers were investigated. Scanning electron microscopy, differential scanning calorimetry, and Fourier-transform infrared spectroscopy (FTIR), as well as cell culturing of MC3T3-E1 were used in this study. The results showed that centrifugal speeds from 350 to 1500 rpm satisfied the needs for fiber formation. The PLA fibers we prepared had three-dimensional structures with extensive diameter distribution from the nanoscale to several tens of micrometers, large pore sizes, and high porosities, significantly different from fibers produced by electrospinning. The fiber diameters and mechanical properties could be manipulated by controlling the centrifugal speed. The finest fibers were generated at 900 rpm with average diameters of 3.47 ± 3.48 μm. The fibers created by centrifugal melt-spinning exhibited lower cytotoxicity and higher cell proliferation than those obtained by electrospinning.

## Abstract 2

Schematic of the centrifugal melt spinning apparatus, consisting of (a) a rotary disk, (b) heating circuit, (c) electromotor, (d) filling inlet and (e) rotary collector on a drum.

## Graphical abstract

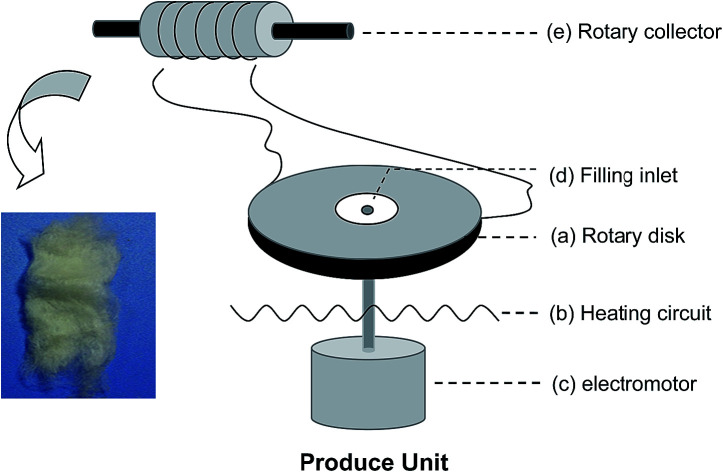

## Introduction

Because they offer large surface-area-to-volume ratios and a wide range of morphologies and geometries in three-dimensional polymeric scaffolds, fiber-based biodegradable materials have attracted increasing attention for many applications in recent years. The past decade has seen increasingly rapid advances in the field of biodegradable polymer fibers employed as tissue engineering scaffolds.^1^ Although most materials in tissue engineering are porous foam scaffolds produced by the particulate leaching method, gas foams, and freeze-drying, the large surface-area-to-volume ratio, flexibility, and high permeability of fibers grant them potential in tissue engineering applications.^2–4^ Electrospinning has been extensively employed as a technique to generate scaffolds for tissue engineering. The nanoscale and nonwoven structures of electrospun fibers, characterized by inherent porosities and random arrangements, can mimic that of extracellular matrices (ECMs), which is necessary for any *in vivo* tissue engineering application. However, the average pore size of such fibers is less than 1 μm, much smaller than the actual cell size of 5–20 μm. Pore sizes below the average cellular diameter block cell migration within the structure. Thus, cells inevitably failed to penetrate electrospun nanofibrous scaffolds.^5^ Moreover, in electrospinning, the residual solvent residing in the fibers can be detrimental to various cellular activities, compared to those produced by solvent-free processes, which restricts the applications of electrospun fibrous scaffolds or mats in tissue engineering applications.^6,7^

Thus, in this study, we attempted to attain larger pore sizes to facilitate infiltration and cellular in-growth, while avoiding solvent usage. Inspired by cotton candy machines, in this work, we manufactured an inexpensive centrifugal spinning system for the high-rate and low-cost synthesis of fibers with diameters ranging from nanometers to micrometers in scale. Based on this system, cotton-like nonwoven fibers of PLA were fabricated successfully. In this study, we focused on the effects of the centrifugal melt-spinning parameters on the diameter distribution of the fibers. In addition, the physical and mechanical properties and cell compatibility of the fibers were studied.

## Materials and methods

### Centrifugal melt-spinning device

**Scheme 1:**
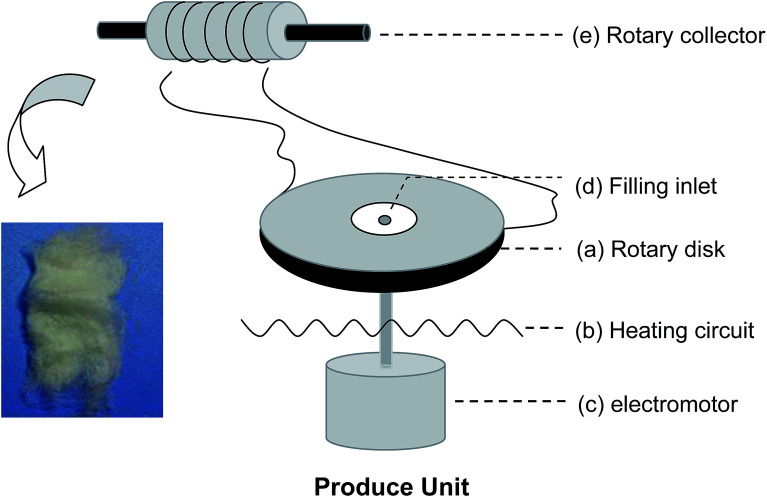
Schematic of the centrifugal melt spinning apparatus, consisting of (a) a rotary disk, (b) heating circuit, (c) electromotor, and other electro-circuit-controlling devices refitted from a cotton candy machine. The centrifugal speed is between 350 and 2000 rpm, according to the electromotor. The temperature of the disk can be controlled from 20 to 300 °C. The polymer powder fills the rotary disk through the filling inlet (d), melts instantly, fuses into fibers under centrifugation at different speeds, and collects on a rotary drum (e).

Scheme 1 shows a schematic of the centrifugal melt-spinning system developed in our present study. The apparatus consists of a rotary disk, heating unit, electromotor, and other electro-circuit-controlling devices refitted from a cotton candy machine. The centrifugal speed is between 350 and 2000 rpm based on the electromotor. The temperature of the disk can be controlled from 20 to 300 °C. The PLA powder fills the heated disk through the filling inlet, melts gradually, and fuses into fibers under centrifugation at different speeds before collection by a rotary drum. PLA with the viscosity-average molecular weight (*M*_v_ = 90 595) was synthesized in our laboratory.

### Preparation of nonwoven PLA fibers

Before processing, the PLA powder was dried at 70 °C for 4 h in a convection oven to avoid hydrolytic degradation during manufacturing. The dried PLA powder (denoted “RAW”) was poured into the heated rotary disk through the filling inlet. According to our undocumented experimental data, a successional and higher yield of PLA fibers could be obtained for the central and edge temperatures of the rotary disk of 220 °C and 180 °C, respectively. The melt temperatures differed, determined by the intrinsic thermodynamic properties of the raw materials. Three kinds of fibers were achieved by different rotary speeds. The speeds were set as 350, 900, and 1500 rpm, denoted as LS, MS, and HS, respectively. The primary fibers (denoted 1-LS, 1-MS, and 1-HS) were gathered by a rotating drum. Then these primary fibers were used as the raw materials to produce secondary fibers under the same speeds by repeating the procedure described above; the products are denoted 2-LS, 2-MS, and 2-HS, respectively. Electrospun PLA fibers were used as a control group; these were produced under conventional conditions (concentration of polymer in chloroform: 8% (w/v); voltage: 20 kV; distance: 15 cm).

### Morphological and diameter characterization

The morphologies of the PLA nonwoven fibers were characterized by digital photography and field-emission scanning electron microscope (FESEM, Philips XL30). The fiber samples were plated with a thin layer of gold before FESEM observation. The FESEM images were analyzed with NIH Image J software (provided by the National Institute of Health, USA) to determine the fiber diameters. 1000 fibers for each group were measured.

### Tensile testing

Mechanical testing of the fibers was performed on an Instron 1121 (US) universal testing machine at room temperature and the relative humidity of 47%. Tensile measurements were performed with the crosshead speed of 5 mm min^−1^ and initial grip separation of 20 mm. In order to ensure the comparability of different samples, the weight and length of the fibers were set as 0.4 g and 3.0 cm uniformly and respectively to ensure that the cross-sectional areas would be 0.095 cm^2^, theoretically. An average of four individual tensile determinations was performed for each sample; the mean and standard deviation of these four determinations are presented. To characterize the fiber structure, the tensile strength, elastic modulus, and stress–strain behaviors were tested on the tensile tester.

### Intrinsic viscosity molecular measurement

The viscosity-average molecular weight (*M*_v_) was measured using gel permeation chromatography (GPC) at 30 °C. The extents of decrease in the *M*_v_ were defined by the differences between the *M*_v_ of the PLA before (*M*_v1_) and after (*M*_v2_) centrifugal melt spinning, *i.e.*,

1 Extent of decrease = ((*M*_v2_ − *M*_v1_)/*M*_v1_) × 100%

### Fourier-transform infrared spectroscopy (FTIR)

In order to identify the chemical structural differences in the fibers, the infrared (IR) spectra was obtained using a Bruker Vertex ×70 Fourier transform IR (FTIR) spectroscope by the viscosity method in a dilute polymer/chloroform solution of 3 mg mL^−1^ PLA.

### Differential scanning calorimetry (DSC)

DSC scans were performed in the temperature range from 0 to 220 °C at a heating rate of 20 °C min^−1^ and the nitrogen flow was set to 50 mL min^−1^ using a DSC Q100 (TA Instrument, USA). From the thermograms, the crystallinities of the samples were recorded using the Universal Analysis 2000 program available from TA Instrument. All measurements were taken from the calorimetric date for the first heating cycle, because the fibers in the second heating scan show no differences.^2^ The degree of crystallinity (*X*_DSC,c_) was estimated considering the ideal melting enthalpy of 93.7 J g^−6^ according to the following equation:

2 *X*_DSC,c_ (%) = 100 × (Δ*H*_m_ − Δ*H*_c_)/93.7

### Cytotoxicity test

The cytotoxicity of the fibers was determined based on the viability of MC3T3-E1 cells in material extracts using a 3-(4,5-dimethylthiazol-2-yl)-2,5-diphenyltetrazolium bromide dye (MTT, Sigma-Aldrich) assay. The fiber specimens from centrifugal melt-spinning and electrospinning were immersed into Dulbecco's Modified Eagle Medium (DMEM) containing 10% serum and incubated at 37 °C for 72 h to obtain the material extracts. The ratio of the fiber specimen to the medium was 0.5 g/25 mL. The material extracts were used as the cell culture media to perform the MTT assay. The MC3T3-E1 mouse pre-osteoblasts were purchased from the Shanghai Institute of Cell Biology, Chinese Academy of Sciences (CAS). The cells were seeded into a 96-well tissue culture plate (TCP, Gibco) at a density of 1 × 10^4^ cells per well and were cultured at 37 °C and 5% CO_2_ under static conditions for up to 24 h. The medium of each well was then replaced with 200 μL of the material extract and the cells were incubated for another 20 h. TCP was set as the control and 6 replicates were used for each group. 20 μL of MTT (5 mg mL^−1^ in phosphate-buffered saline (PBS)) was added to each well, after which the cells were incubated for an additional 4 h. The medium was then removed and 150 μL of dimethyl sulfoxide (DMSO) was added to each well to solubilize the converted dye. The absorbance values at 450 nm were measured on a multifunctional microplate scanner (Tecan Infinite M200).

### Cell proliferation and cell morphology

The cellular attachment and cell viability of the fibers were determined using the MTT assay. The fibers and electrospun specimens were filled into a 48-well plate at 0.15 g per well. Before seeding, the fibers were pre-wetted by submersion in a 70% aqueous ethanol solution overnight and then immersion in media for 2 h in an incubator at 37 °C.^7,8^ After 1, 3, 7, and 14 days of incubation, 100 μL of MTT was added and incubation was continued for 6 h. 750 μL of acidified isopropanol (0.4 mol L^−1^) was added to each well and incubated at 37 °C for 15 min to solubilize the converted dye. The solution in each well was mixed 200 μL samples were obtained and transferred to another 96-well plate. The optical densities were measured at 540 nm wavelength on a multifunctional microplate scanner (Tecan Infinite M200). TCP was set as the control and three replicates used for each group.

A morphological study of the MC3T3-E1 cells cultured on different samples was performed after 14 days of cell culturing, using FESEM. The cell–fiber constructs were rinsed three times with PBS and fixed in 3% glutaraldehyde for 24 h. The samples were further rinsed in PBS and dehydrated with increasing concentrations of ethanol (50%, 60%, 70%, 80%, 90%, and 100%) for 30 min each. Finally, the cell–fiber constructs were freeze-dried for 2 d before FESEM observation.

### Statistical analysis

All experiments were performed in triplicate at the minimum. All quantitative data is expressed as the mean ± the standard deviation. Wherever appropriate, comparisons of the means were performed using Origin 8.0 software (OriginLab Corporation, USA), with *p* < 0.05 considered statistically significant.

## Results and discussion

### Morphology and diameters

**Fig. 1:**
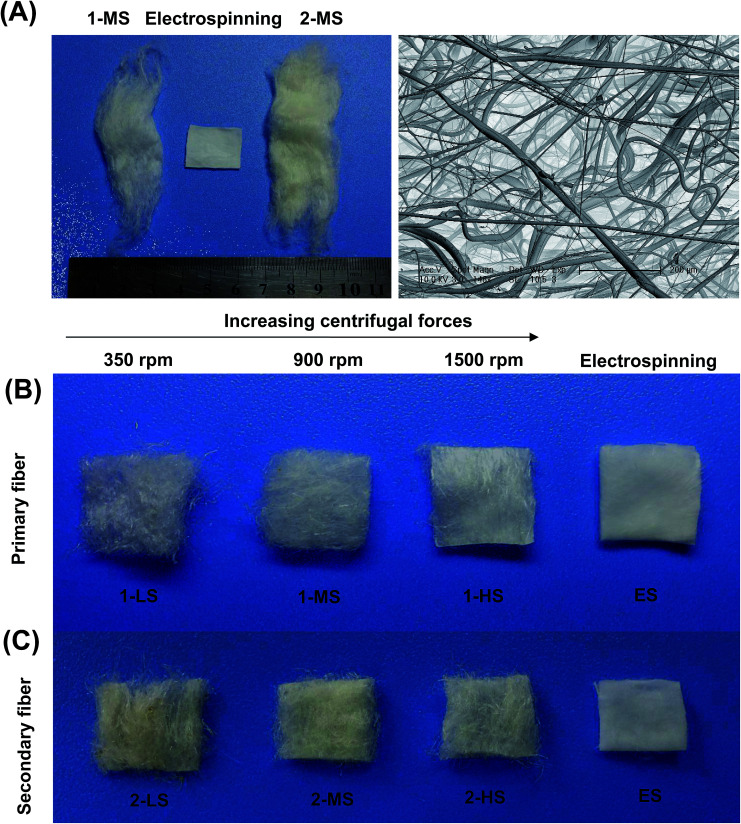
The appearances of PLA nonwoven fibers fabricated by centrifugal melt spinning compared to electrospun fibers: (A) 1-MS primary and 2-MS secondary fibers, left and right, from melt spinning at medium speed (900 rpm), and electrospun fibers, middle; (B) primary fibers formed at speeds of 350 (1-LS), 900 (1-MS), and 1500 (1-HS) rpm; (C) secondary fibers (2-LS, 2-MS, and 2-HS) at same speeds. All samples shown in (B) and (C) are approximately 100 mm^2^. The pictures were taken by a digital camera.

**Fig. 2:**
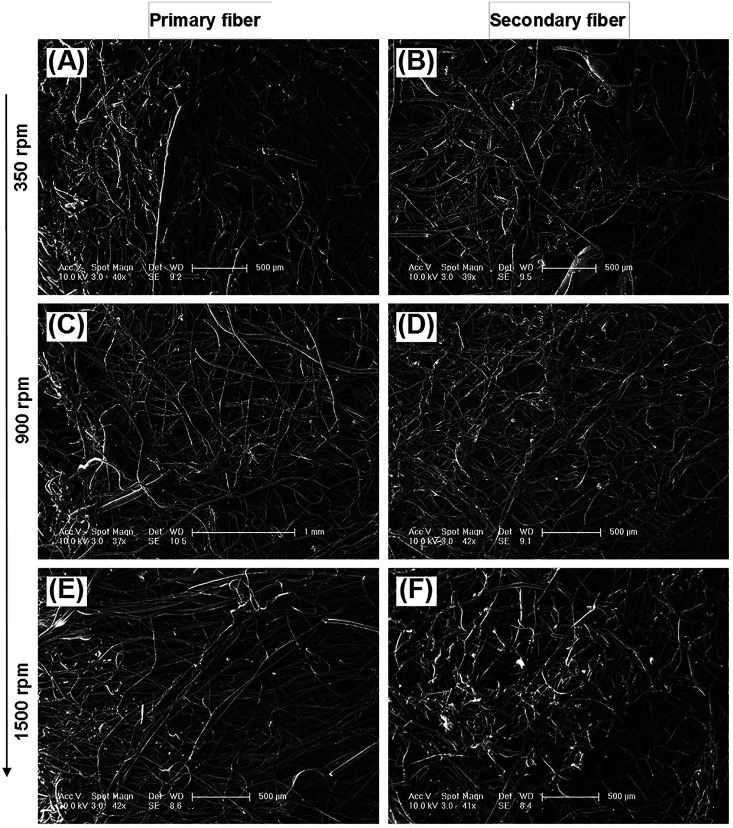
Low-magnification SEM images of centrifugal melt-spun nonwoven PLA fibers obtained at different rotation speeds: (A and B) low, (C and D) mid, and (E and F) high. (A), (C), and (E) Show primary fibers; (B), (D), and (F) show secondary fibers. The scale bars are 500 μm for (A), (B), (D), (E), and (F), and 1 mm for (C).

**Fig. 3:**
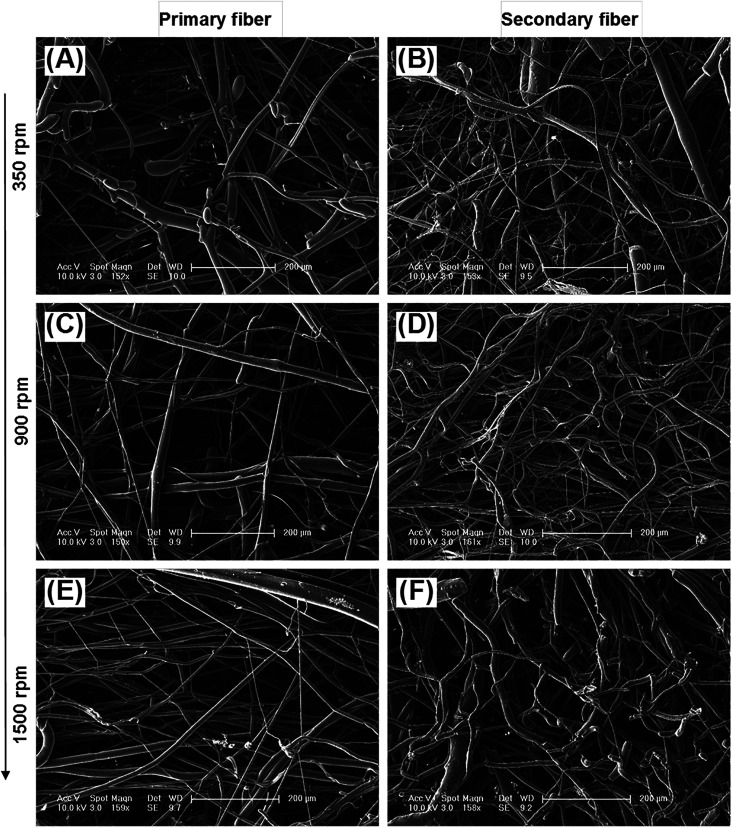
High-magnification SEM images of centrifugal melt-spun nonwoven PLA fibers fabricated at different rotation speeds: (A and B) low, (C and D) mid, and (E and F) high. (A), (C), and (E) Depict primary fibers; (B), (D), and (F) show secondary fibers. All scale bars are 200 μm.

The diameter distribution, pore size, and orientation of fibers are critical for tissue engineering applications.^8^ PLA is one of the most promising biodegradable polymers in tissue engineering; PLA fibers prepared by different methods, such as melt spinning,^9–15^ solution spinning,^16,17^ and electrospinning,^18–21^ have been reported. In this work, we manufactured a centrifugal melt-spinning system for fabricating nonwoven fibers of PLA. The morphologies of the fibers, observed by optical microscopy and FESEM analysis, are shown in Fig. 1–3.

Fig. 1 shows that the nonwoven PLA fibers formed by the centrifugal melt spinning method are loose and cotton-like, significantly different from the flake of electrospun PLA fibers. For the primary melt-spinning process (Fig. 1B), the fibers of 1-LS, fabricated at a low rotation speed, are rough and non-homogeneous. The fineness and homogeneity of the primary fibers is increased gradually as the centrifugal speed increases. However, no similar tendency appears in the fiber morphology for the secondary melt-spinning process (Fig. 1C). The 2-MS fibers appear finer and more homogeneous than the other two secondary melt-spinning samples. Low-magnification FESEM observation shows that the 1-HS and 2-MS fibers exhibit the greatest fineness and homogeneity (Fig. 2), which is consistent with the optical observations. Furthermore, the high-magnification FESEM observations indicate that the fibers form a reticular and interconnected three-dimensional structure (Fig. 3). By centrifugal melt spinning, desirable three-dimensional structures can be formed, but similar structures are difficult to obtain by electrospinning.

**Fig. 4:**
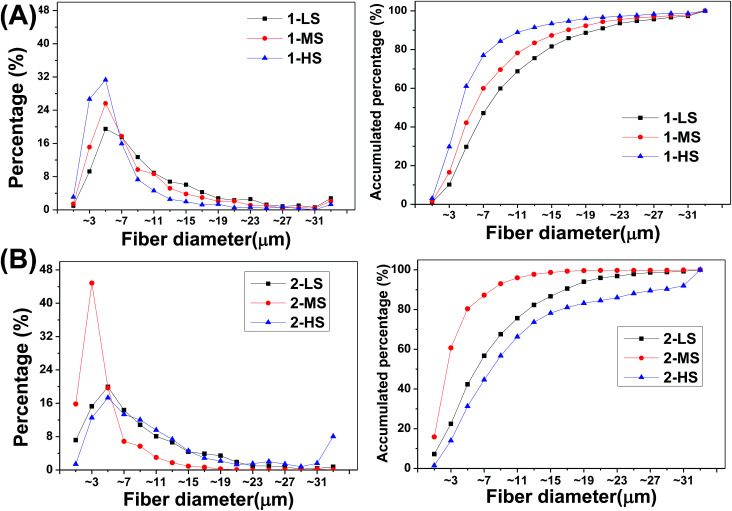
(Left) Frequency distributions and (right) accumulated frequency distributions of fiber diameters for (A) primary and (B) secondary fibers.

The size distributions of the different fibers are analyzed as shown in Fig. 4 and Table 1.

**Table 1:** Diameter distributions of different fibers

Sample	Min.–maximum	Quartile (*Q*_1_–*Q*_3_)	Median	Mean
1-LS	0.10–101.61	4.58–12.91	7.43	9.98 ± 8.73
1-MS	0.12–72.22	3.62–9.99	5.74	8.27 ± 7.93
1-HS	0.15–67.33	2.71–6.65	4.15	5.99 ± 6.11
2-LS	0.17–61.51	3.26–10.81	5.93	7.89 ± 6.76
2-MS	0.1 0–33.39	1.41–4.23	2.37	3.47 ± 3.48
2-HS	0.12–178.07	4.23–13.44	7.66	12.98 ± 16.95

For the primary fibers (Fig. 4), the diameters of 1-LS, 1-MS, and 1-HS have ranges of 0.10–101.61 μm, 0.12–72.22 μm, and 0.15–67.33 μm, with median diameters of 7.43, 5.74, and 4.15 μm, respectively. This indicates a negative correlation of fiber diameter with rotation speed from 350 rpm to 1500 rpm. The fibers formed at the highest rotation speed show the lowest diameters and the highest central peak. However, for the secondary fibers, the 2-MS fibers formed at the mid-range speed of 900 rpm show the finest diameters and the highest central peak in the diameter distribution (Fig. 4B). The 2-MS fiber diameter ranges from 0.10 to 33.39 μm with a median diameter of 2.37 μm, slightly more than half the 1-HS diameter at 4.15 μm. The 2-MS fibers show 80.4% of fibers having diameters between 0 and 6 μm, which is significantly different from the other distributions. The median diameter of the 2-LS fibers is 5.93 μm, similar to the 5.74 μm diameter of the 1-MS fibers. Meanwhile, at the increased rotation speed of 1500 rpm, the diameters of the 2-HS fibers are increased. The diameter distribution of the 2-HS fibers does not follow the tendency of increasing diameter with increasing centrifugal speed. Because the viscosity of the spinning fluid is positively associated with the crystallinity and the amount of chain scission, lower-viscosity precursor fluids facilitate PLA fiber formation, which supports the smaller diameters of 2-LS and 2-MS relative to the primary ones. However, in preparing the 2-HS fibers, the balance between the centrifugal force and viscosity of the PLA is broken; thus, the less viscous PLA fluid cannot resist the strong centrifugal force produced by the high centrifugal speed, and therefore, 2-HS contains coarser fibers. In short, based on these, we can fabricate ideal nano- and microfibers with various diameters. The physical and chemical properties could be adjusted by varying the centrifugal speed and raw material.

The centrifugal melt-spun fibers have more extensive fiber diameter distributions, ranging from single nanometers to dozens of micrometers, unlike the narrow nanoscale distribution of electrospun fibers (the measurement of nanoscale fibers was handicapped by technology restrictions). Two techniques of electrospinning and needle punching have been used to combine the beneficial properties of nanofibers and microfibers in three-dimensional porous structures.^21^ However, in this study, we fabricated nano- and microfibers using a single technique. In other words, it was easier in preparation process to obtain nano- and microfibers by this method. In addition, the pore sizes of the fibers are clearly larger than the size of cells, and the fiber orientation is three-dimensional and interconnected.

The diameter distribution of fibers is an important surface morphology parameter of scaffolds for cell contact, affecting cell proliferation and differentiation.^22,23^ Takahashi *et al.* found that the number of mesenchymal stem cells (MSCs) attached to non-woven fabrics was increased with increasing fiber diameter and that both the alkaline phosphatase (ALP) activity and the osteocalcin content of MSCs, as bone differentiation markers, peaked for fiber diameters of ∼9–12 μm.^8^ Nanoscale fibers have been widely used for tissue engineering because they can mimic the nanoscale geometry and topology of ECM structures. We find that fibers with single diameter distributions are unable to meet many tissue-engineering needs. According to our calculations, the advantages of both nano- and microscale structures can be provided by a wide diameter distribution, with new advantages in addition. For example, scaffolds composed of fibers with wide diameter distributions may show better permeabilities than those of nanoscale fibers as well as higher surface areas than those of microscale fibers, both of which facilitate gradual tissue ingrowth. Moreover, the loose compact nanoscale networked structure mimics ECMs, while microscale fibers provide the mechanical support. Therefore, the PLA fibers prepared in this study should have much potential in tissue engineering applications because they have broad diameter distributions ranging from nanometers to dozens of micrometers that can be adjusted by changing the centrifugal speed. Using such fibers, we have fabricated three-dimensional scaffolds for bone tissue engineering in our laboratory.^24–26^

### Mechanical properties

**Fig. 5:**
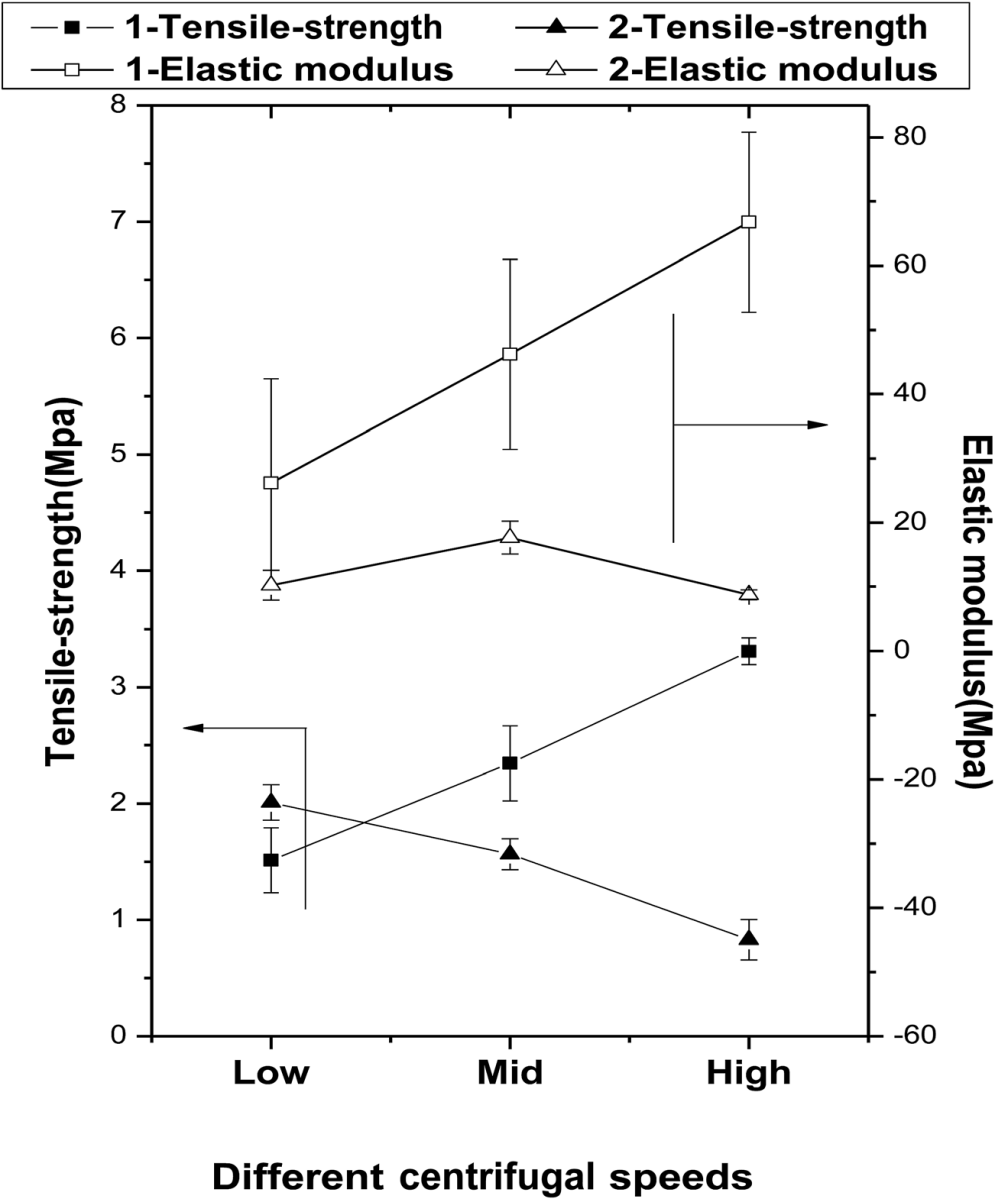
The effects of centrifugal speeds and secondary melt processing on the mechanical properties of the fibers.

The mechanical properties of the fibers fabricated at rotation speeds of 350, 900, and 1500 rpm are presented in Fig. 5.

The tensile strengths of the primary fibers are increased significantly with increasing centrifugal speed (1.51 ± 0.28, 2.34 ± 0.32, and 3.31 ± 0.12 MPa for 1-Ls, 1-MS, and 1-HS, respectively), and their elastic moduli follow the same trend (1-Ls < 1-MS < 1-HS). However, among the secondary fibers, the tensile strength of 2-LS is 2.01 ± 0.15 MPa, but those of 2-MS and 2-HS are lower. Under the same centrifugal speeds, the tensile strengths of 2-MS and 2-HS are clearly smaller than those of 1-MS and 1-HS (Fig. 5 and Table 2).

**Table 2:** The mechanical properties of the different fibers

	LS		MS		HS	
	1-LS	2-LS	1-MS	2-MS	1-HS	2-HS
Tensile stress (MPa)	1.51 ± 0.28	2.01 ± 0.15	2.34 ± 0.32	1.56 ± 0.13	3.31 ± 0.1	0.83 ± 0.17
Elastic modulus (MPa)	26.18 ± 16.20	10.23 ± 2.33	46.21 ± 14.78	17.65 ± 2.58	66.77 ± 14.04	8.75 ± 0.76
Stress–strain (%)	7.77 ± 2.3	10.3 ± 1.0	8.47 ± 0.8	11.67 ± 1.1	7.47 ± 2.5	12.33 ± 1.5

The elastic moduli of the secondary fibers are 10.23 ± 2.33 MPa, 17.65 ± 2.58 MPa, and 8.75 ± 0.76 MPa, significantly lower than those of the primary fibers.

The biomechanical properties of scaffolds are essential to support the attachment, proliferation, and differentiation of cells. In this study, the primary fibers have higher tensile strengths and elastic moduli than the secondary fibers; however, the secondary fibers have greater strain capacities. We attribute this to the crystallinity reduction during the secondary melt-spinning process and the viscosity-average molecular weight reduction by random chain scission reactions, intermolecular and cyclic oligomerization, and/or transesterification, as confirmed later by FTIR. The dramatic reduction in crystallinity during the melt-spinning process is a significant but inevitable defect of this preparation method, because the process requires high temperatures.

### Thermal degradation during melt spinning

Table 3 shows that the viscosity-average molecular weights *M*_v_ of the primary fibers are decreased by approximately 12.69%, 8.59%, and 1.85% compared to the raw material, respectively. Simultaneously, the *M*_v_ of the secondary fibers are decreased by approximately 22.28%, 16.41%, and 5.88% compared to the raw material and by 10.98%, 8.55%, and 4.11% compared to the primary fibers.

**Table 3:** The viscosity-average molecular weights (*M*_v_) and crystallinities of different fibers compared to the raw material

	RAW	LS		MS		HS	
		1-LS	2-LS	1-MS	2-MS	1-HS	2-HS
*M*_v_	90 595	79 096	70 412	82 809	75 730	88 918	85 267
(*M*_v0_ − *M*_vs_)/*M*_v0_ × 100%	—	12.69	22.28	8.59	16.41	1.85	5.88
(*M*_v1_ − *M*_v2_)/*M*_v1_ × 100%		—	10.98	—	8.55	—	4.11
Crystallinity (%)	71.6 ± 1	8 ± 0.3	2.3 ± 0.4	9.2 ± 0.4	5.6 ± 0.4	15.5 ± 0.5	13.7 ± 0.4

**Fig. 6:**
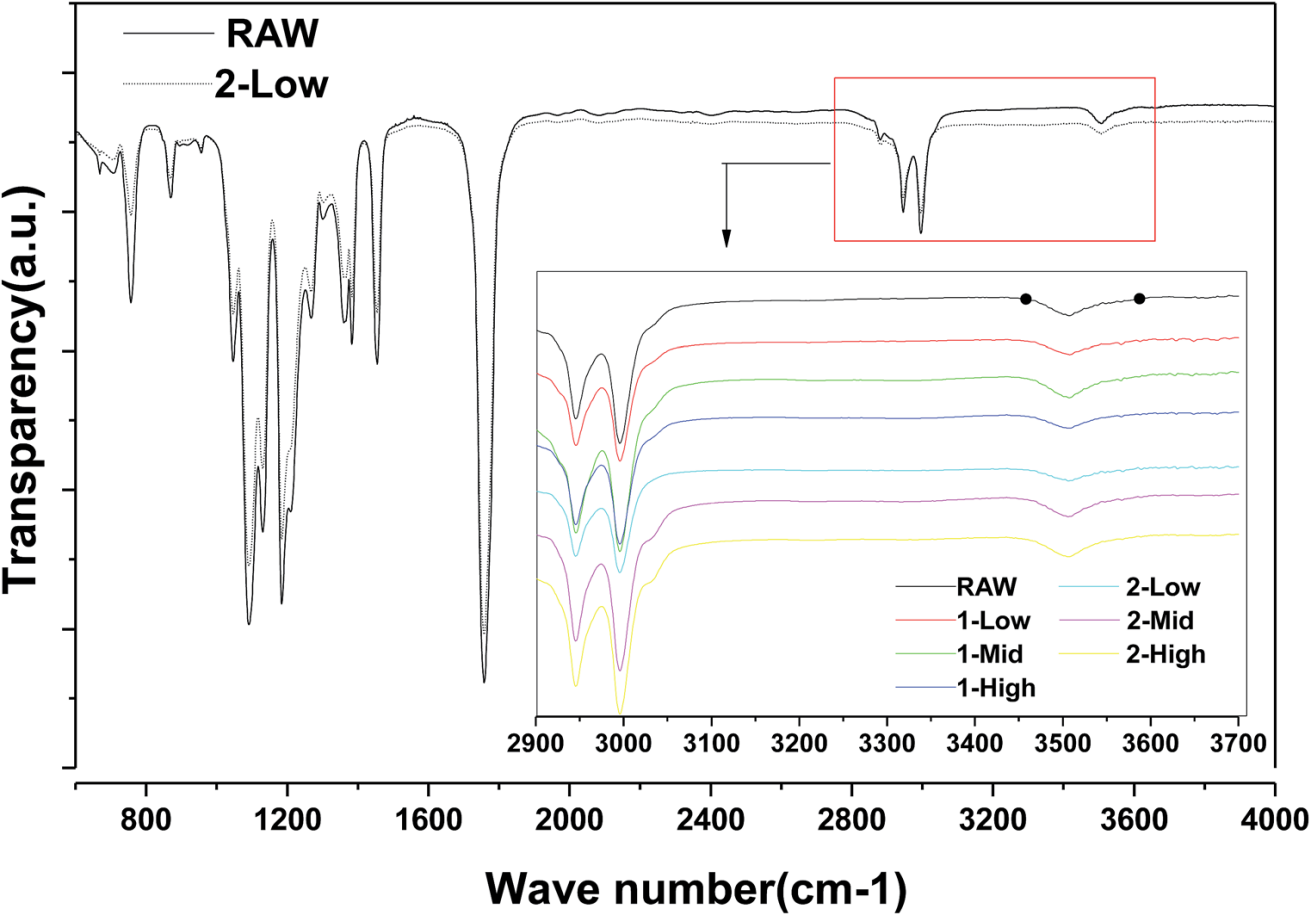
FTIR spectra of raw material and different centrifugal melt spinning fibers.

As shown in Fig. 6, FTIR analysis indicates no obvious differences between the raw material and 2-LS fibers; no new hydroxyl groups, which would be the products of ester hydrolysis in all fibers, are formed from carboxylic acid and alcohols.

The thermal decomposition and stability are important PLA fiber properties determining the reprocessing performance and degradation of the final materials. The decreases in viscosity-average molecular weight show tendencies similar to that of the thermal stability. Higher centrifugal speeds correspond to faster PLA cooling and shorter durations in the heated disk.

### DSC

**Fig. 7:**
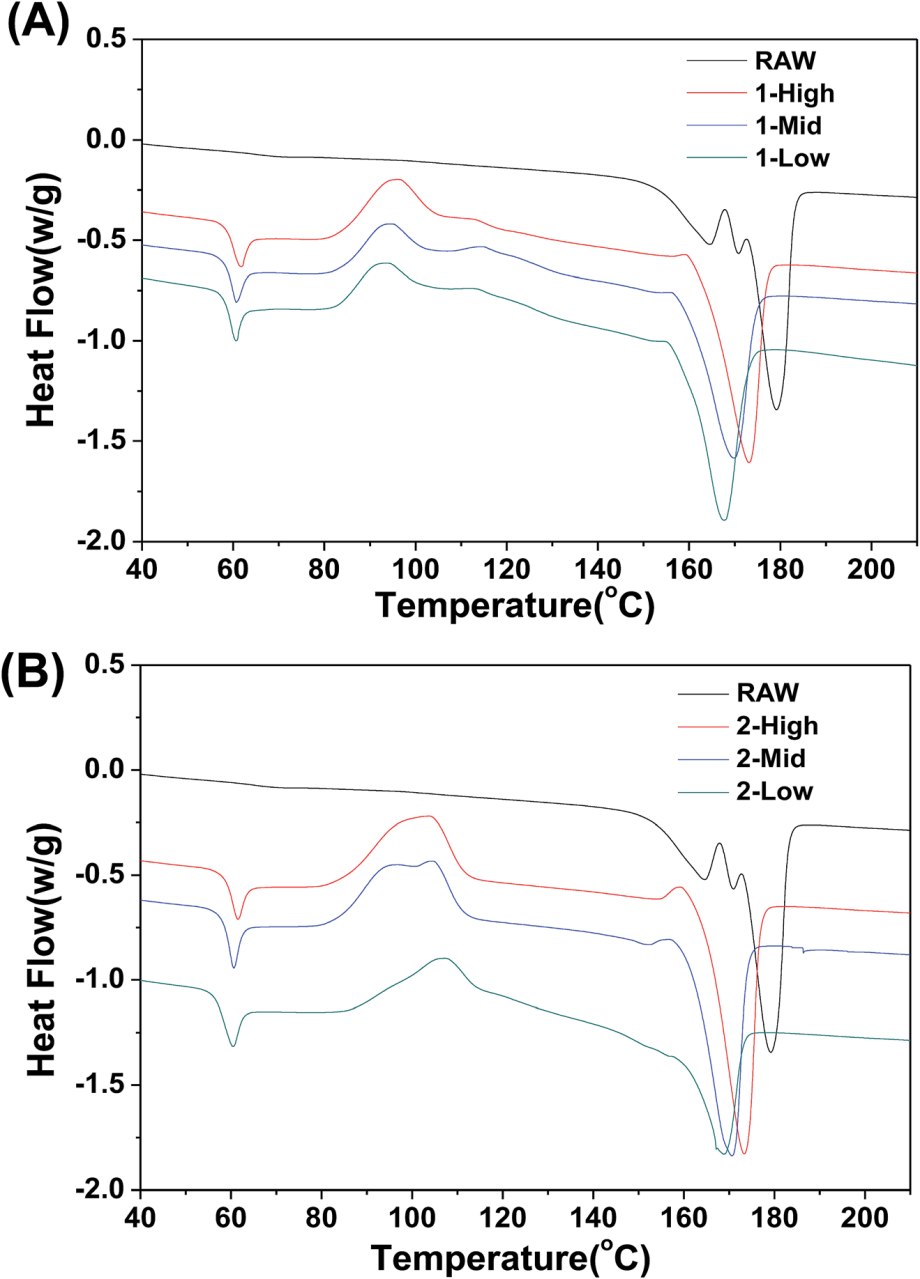
DSC thermograms of the first heating cycles of (A) primary and (B) secondary fibers.

The DSC thermal properties of the fibers are shown in Fig. 7. The thermal transitions of the fibers are important to determine the processing and end-use treatment of products containing them. In contrast to the RAW material, the melt-spun PLA fibers show greater variation in thermal transition. The DSC thermogram of the fibers shows *T*_g_ values of ∼60 °C irrespective of fiber formation phase (Fig. 7A and B). An exothermic peak representing cold crystallization (*T*_c_) is observed between 83 °C and 90 °C, and the melt temperature (*T*_m_) is shifted to lower temperatures between 159 °C and 165 °C. Unlike *T*_g_ and *T*_c_, *T*_m_ decreases slightly as the spinning speed is decreased. As the speed increases, the fiber crystallinity increases, which elevates the melting point.

### Cytotoxicity and cell viability

**Fig. 8:**
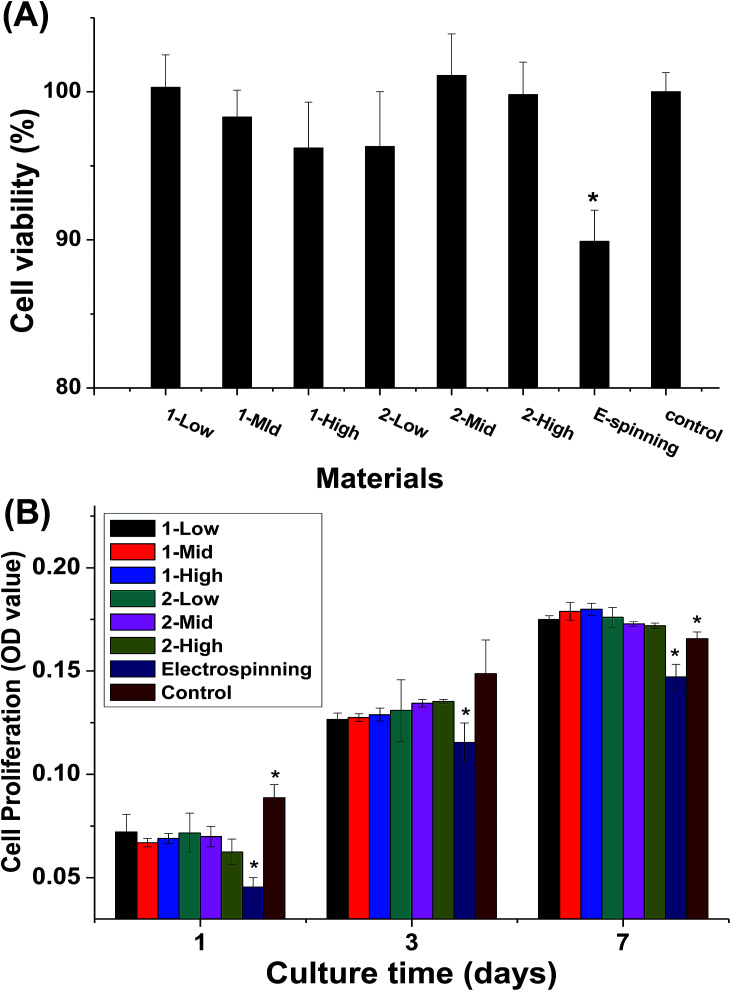
MTT assay results of centrifugal melt-spun PLA fibers: (A) cell cytotoxicity of obtained fibers and (B) cell proliferation on the fibers for 1, 3, and 7 days. Asterisks (*) indicate statistical significance *p* < 0.05.

The cytotoxicity of the material extracts and the cell proliferation of MC3T3-E1 cells cultured on the fibers are shown in Fig. 8. The melt-spun fibers show no significant differences in cytotoxicity, but they are less cytotoxic than the electrospun specimens (Fig. 8A). During the first week of culturing, no significant difference is observed in cell proliferation between the melt-spun fibers and control (TCP), but proliferation is higher for the melt-spun than the electrospun fibers (Fig. 8B). The effect of fiber diameter in the fabric constituting the scaffold on the cell behavior has been examined by multiple studies.^3–5^ In this study, the biocompatibility of the melt-spun fibers was assessed by examining their potential to support the growth and proliferation of MC3T3-E1 in the fibers. Since the melt-spun fibers are produced by a solvent-free process, the route is more eco-friendly and less toxic than that using electrospun specimen, as identified by the results of the cytotoxicity analysis. Theoretically, smaller fiber diameter distributions correspond to larger surface areas, which can provide greater cell permeation. Surprisingly, the cell proliferation rates among the fibers show no significant differences, possibly because the pores of the crude fibers are more conducive to cell ingrowth. This assumption can also explain the high cell proliferation rate on the melt-spun fibers after 14 days of culturing, compared to TCP. The results here differ from those reported by Takahashi *et al.*^8^ In their study, MSC was demonstrated to attach, proliferate, and differentiate on PET non-woven fabrics with various diameters and porosities. However, the species and diameter distributions of the fibers differ, making the results incomparable.

### Cell morphology

**Fig. 9:**
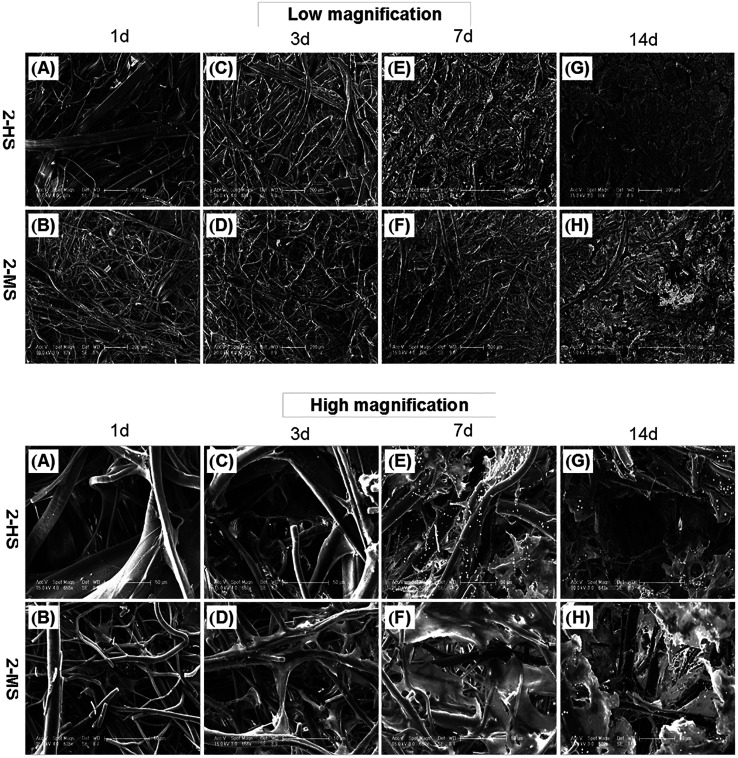
FESEM images of MC3T3-E1 cells grown on (A, C, E, and G) 2-MS and (B, D, F, and H) 2-HS fibers for 1, 3, 7, and 14 days: (A and B) LS, (C and D) MS. The scale bars for low- and high-magnification micrographs are 100 μm and 50 μm, respectively.

As shown in Fig. 9, the morphologies of the MC3T3-E1 cells grown on the 2-MS and 2-HS fibers are analyzed using FESEM. 2-MS and 2-HS were selected for cell morphology analysis because the differences in diameter were the most obvious and the molecular measurements and thermodynamic properties were the most similar between 2-MS and 2-HS. The effect of cell morphology on the fibers was thus entirely determined by the structure of the PLA fiber itself, and the main factor of variation was the fiber diameter distribution. As shown in Fig. 9, MC3T3-E1 cells are attached and spread on all samples; cells on the melt-spun fibers are oriented along the fine fibers and stepped over the coarse fibers after 14 days of culture. More cells and ECM are observed in the 2-HS fibers than in the 2-MS fibers after both 7 and 14 days. All the 2-MS and 2-HS fibers are completely covered by ECM secreted by the cells after 14 days of incubation. One inherent limitation of electrospinning is the relatively poor cellular infiltration into the depth of the scaffold due to small pore size and high fiber packing densities.^27^ The novel method of centrifugal melt spinning was developed in this study to fabricate biodegradable polymer fibers with a diameter distribution of 1–30 μm. Specifically, the pore sizes and orientations of the centrifugal melt-spun fibers are advantageous for cell penetration of the fiber scaffolds.

## Conclusions

Solvent-free PLA nonwoven fibers with different diameter distributions and three-dimensional intersecting structures were prepared by a centrifugal melt-spinning method. The centrifugal melt spinning parameters were investigated to optimize the physical and chemical properties of the fibers. The melt-spun fibers were cotton-like with broad diameter distributions and interconnected three-dimensional structures. The physical and chemical properties could be adjusted by varying the centrifugal speed and raw material. The variety of achievable physical and chemical properties, particularly in the diameter distributions, pore sizes, and intersecting structures, would be useful for tissue-engineering scaffold applications. In addition, the centrifugal melt device can be applied to both dry and wet spinning, indicating significant potential. The results of cell experiments suggested that the fibers produced by our method have lower cytotoxicity and greater proliferation than the electrospun specimens do, although no significant differences appeared among the various centrifugal melt-spun fibers. The cell differentiation properties of the centrifugal melt-spun fibers and the optimized production process remain for further investigation.

## Conflicts of interest

There are no conflicts to declare.

## Supplementary Material
