# Correction: Cotton-like micro- and nanoscale poly(lactic acid) nonwoven fibers fabricated by centrifugal melt-spinning for tissue engineering

**DOI:** 10.1039/c8ra90010h

**Published:** 2018-02-12

**Authors:** Hongli Zhou, Yufeng Tang, Zongliang Wang, Peibiao Zhang, Qingsan Zhu

**Affiliations:** Department of Orthopedics, China-Japan Union Hospital, Jilin University 126 Xiantai Street Changchun 130033 PR China zhuqs@jlu.edu.cn +86 431 85262058 +86 431 85262058; Key Laboratory of Polymer Ecomaterials, Changchun Institute of Applied Chemistry, Chinese Academy of Sciences Changchun 130022 PR China zhangpb@ciac.ac.cn; Department of Traumatology, Qianfoshan Hospital of Shandong Province 250000 PR China

## Abstract

Correction for ‘Cotton-like micro- and nanoscale poly(lactic acid) nonwoven fibers fabricated by centrifugal melt-spinning for tissue engineering’ by Hongli Zhou *et al.*, *RSC Adv.*, 2018, **8**, 5166–5179.

The funding information provided for this paper was incorrect: one funder number was in error while another funder was inadvertently omitted. The revised Acknowledgements section should read as follows:

This research was financially supported by the National Natural Science Foundation of China (No. 51473164, 51403197 and 51673186), the Program of Scientific Development of Jilin Province (20170520121JH and 20130201005GX), the Ministry of Science and Technology of China (International Cooperation and Communication Program 2014DFG52510), the Joint FundeProgram of the Chinese Academy of Sciences and Japan Society for the Promotion of Science (GJHZ1519), Industrial Innovation Special Fund Project of Jilin Province (2018C005), and the Special Fund for Industrialization of Science and Technology Cooperation between Jilin Province and Chinese Academy of Sciences (2017SYHZ0021).

The Royal Society of Chemistry apologises for these errors and any consequent inconvenience to authors and readers.

## Supplementary Material

